# Capsid-Targeted Viral Inactivation: A Novel Tactic for Inhibiting Replication in Viral Infections

**DOI:** 10.3390/v8090258

**Published:** 2016-09-21

**Authors:** Xingcui Zhang, Renyong Jia, Jiakun Zhou, Mingshu Wang, Zhongqiong Yin, Anchun Cheng

**Affiliations:** 1 Avian Disease Research Center, College of Veterinary Medicine of Sichuan Agricultural University, Wenjiang District, Chengdu 611130, Sichuan Province, China; zhangxc923@163.com (X.Z.); zjk_Ailsa@163.com (J.Z.); mshwang@163.com (M.W.); 2 Key Laboratory of Animal Disease and Human Health of Sichuan Province, Wenjiang District, Chengdu 611130, Sichuan Province, China; yinzhongq@163.com; 3 Institute of Preventive Veterinary Medicine, Sichuan Agricultural University, Wenjiang District, Chengdu 611130, Sichuan Province, China

**Keywords:** Capsid-targeted viral inactivation, antiviral strategy, core protein, degradative enzyme, fusion proteins

## Abstract

Capsid-targeted viral inactivation (CTVI), a conceptually powerful new antiviral strategy, is attracting increasing attention from researchers. Specifically, this strategy is based on fusion between the capsid protein of a virus and a crucial effector molecule, such as a nuclease (e.g., staphylococcal nuclease, Barrase, RNase HI), lipase, protease, or single-chain antibody (scAb). In general, capsid proteins have a major role in viral integration and assembly, and the effector molecule used in CTVI functions to degrade viral DNA/RNA or interfere with proper folding of viral key proteins, thereby affecting the infectivity of progeny viruses. Interestingly, such a capsid–enzyme fusion protein is incorporated into virions during packaging. CTVI is more efficient compared to other antiviral methods, and this approach is promising for antiviral prophylaxis and therapy. This review summarizes the mechanism and utility of CTVI and provides some successful applications of this strategy, with the ultimate goal of widely implementing CTVI in antiviral research.

## 1. Introduction

Since the proposal of “intracellular expression of antibodies” [1], the protein-based antiviral strategy capsid-targeted viral inactivation (CTVI) [2] has emerged. This approach is based on incorporation of a foreign gene into a capsid gene. For example, a degradative enzyme is fused to a capsid component to form a capsid–enzyme fusion protein. The fusion protein is then assembled inside the virion, where the capsid–enzyme comes into contact with and destroys viral nucleic acid [3,4,5], resulting in the inhibition of viral replication and a reduction in virus particles [6]. In this strategy, it is essential that the capsid–enzyme fusion satisfies the following requirements: stable expression in transfected cells, synchronous assembly of recombinant proteins in the virion, retention of the original activity (nucleic acid degradation or interference of viral protein folding) of the foreign molecule, and, most importantly, no cytotoxicity.

Thus far, CTVI has been successfully applied for several viruses, such as murine leukaemia virus (MLV) [7,8], classical swine fever virus (CSFV) [9,10], human immunodeficiency virus-1 (HIV-1) [11], Japanese encephalitis virus (JEV) [12], hepatitis B virus (HBV) [13,14], and dengue 2 virus (DENV2) [15,16,17] (Table 1). Herein, we summarize the current knowledge about the mechanism and application of CTVI, with the purpose of providing a new approach for antiviral prophylaxis and therapy.

## 2. Mechanisms of CTVI

**Figure 1:**
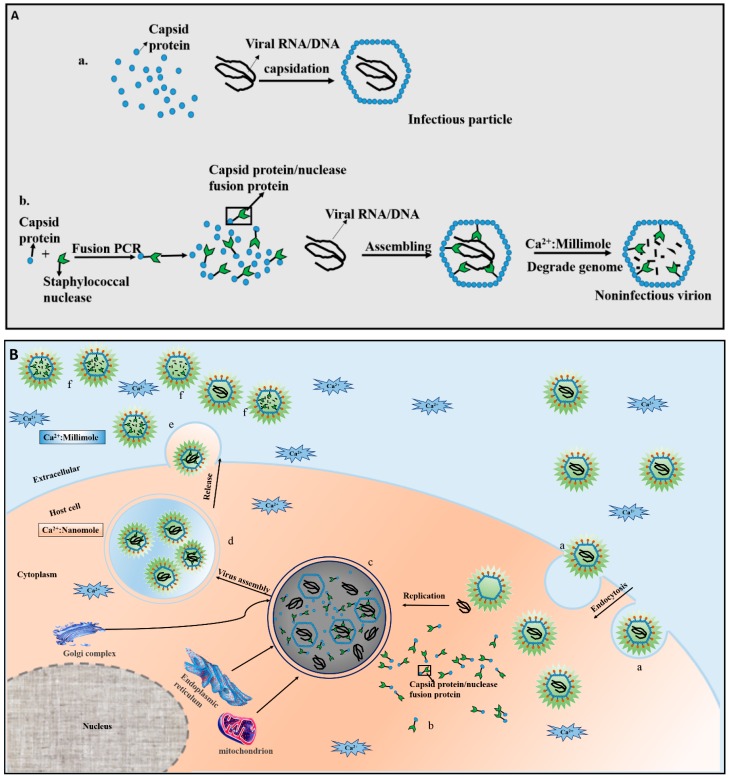
The main mechanism of capsid-targeted viral inactivation (CTVI). (**A**) Comparison of viral genome encapsidation in a normal virus and a virus containing a capsid–enzyme fusion protein [3]. **a**. Normal assembly of viruses, whereby nucleic acid is enveloped by capsid proteins to form the nucleocapsid; **b**. The assembly process of the virus with a fusion protein composed of a capsid protein and a degradative enzyme (e.g., staphylococcal nuclease (SN)) that is calcium ion (Ca^2+^) dependent. The fusion protein is incorporated into the internal virion during viral assembly, where is has direct access to nucleic acid. When the Ca^2+^ concentration reaches the millimolar range, the enzyme is activated and digests the viral RNA/DNA; (**B**) A schematic representation of the CTVI mechanism. Infection of a cell by a virus stably expressing a fusion protein mainly includes the following steps: **a**. The virus enters the host cell through the endocytosis pathway; **b**. the fusion protein is stably expressed; **c**. using material supplied by the host cell, the fusion protein is incorporated into the viral structure during viral assembly; **d**. the virus is assembled and modified to form a mature virion within closed vesicles in the cytoplasm, but the nuclease in the virion is inactive due to the intracellular nanomolar Ca^2+^ concentration; **e**. the virus is released into the extracellular environment; **f**. the nuclease incorporated into the progeny virion is active in the extracellular millimolar Ca^2+^ concentration, where it can degrade the viral nucleic acids.

The strategy proposed by Natsoulis and Boeke [3] in 1991 was first used in a model of yeast retrotransposon Ty1, and the method has since been applied to various viruses. The mechanism underlying this strategy is based on the fact that viral DNA or RNA is encapsidated into a protein shell, forming a complex. The complex, comprising capsid proteins and nucleic acid, is termed the nucleocapsid (Figure 1Aa). As the protein of the nucleocapsid participates in viral assembly, a fusion protein consisting of a virus nucleocapsid protein and a foreign protein (an effector such as a nuclease [26,27], lipase, protease, or single-chain antibody (scAb) [12,20,28] is generated. The fusion protein is then incorporated into the virus particle. Therefore, the effector molecule of the fusion protein has direct access to the nucleic acid or protein components of viruses. In particular (Figure 1Ab), the fusion protein can degrade DNA/RNA [29,30] or disrupt the protein composition of the virus [21], resulting in an antiviral effect.

The foreign molecule chosen for CTVI should be nontoxic to cells. Herein, we utilize staphylococcal nuclease (SN) [31] for describing the mechanism of CTVI. SN is an exotoxic protein produced by *Staphylococcus aureus* [32,33] that is strictly calcium ion (Ca^2+^) dependent, requiring approximately 0.5–1 millimolar amounts of Ca^2+^ [34,35]. However, as the concentration of Ca^2+^ in most eucaryotic cells is within the nanomolar range [36], SN is inactive intracellularly and can degrade neither viral nor cellular nucleic acid. Accordingly, the enzyme is not cytotoxic. Conversely, blood and other bodily fluids contain millimolar concentrations of Ca^2+^ [12]. Therefore, when viruses are released into the extracellular environment, SN recovers activity due to the high concentration of Ca^2+^, and SN can degrade the viral genome. (Figure 1B). Nonetheless, not all enzymes are alike. For example, *Serratia marcescens* nuclease (SMN), the activity of which requires magnesium ion (Mg^2+^) [37], is extremely toxic to host cells [38]. Therefore, SMN is not an ideal candidate for CTVI.

In the CTVI strategy, a fusion gene should be constructed in which a portion encodes the capsid protein and a portion encodes the foreign protein. The foreign gene is fused to the C-terminus of the capsid gene because the C-terminus of the encoded fusion protein contacts the viral nucleic acid inside the virion, whereas the N-terminus faces outward [4]. The fusion gene is inserted into a corresponding vector for transfection into susceptible cells, and the fusion protein is expressed. Capsid virus-like particles are formed by the fusion protein during encapsidation of the viral genome. The nuclease is then targeted towards the internal viral particles and is activated to degrade the genome, thereby interfering with generation of the viral genome and ultimately affecting the viral life cycle. Theoretically, the nuclease can efficiently disable virus particles even when only one nuclease molecule is incorporated into the virion [10].

In comparison with an enzyme, there are a few differences regarding the mechanism for scAbs. The scAb portion of the fusion protein interacts with and binds specifically to a C-terminal part of a key viral protein, such as integrase, and thereby disturbs the proper folding of an important protein to inhibit integration and block replication when expressed intracellularly before infection, reducing the infectivity of progeny viruses [20]. scAb is an excellent fusion partner because it can be efficiently incorporated along with its fusion partner into virus particles and bind specifically to viral integrase. Indeed, compared with enzymes, it may be an ideal antiviral molecule for inhibiting the replication of viruses without remarkable cell toxicity [39]. Regardless, a limitation of scAbs is that they can only be applied to viruses that express a relevant protein, such as integrase.

CTVI mainly refers to the capsid targeting of a certain molecule that interferes with the replication of retroviruses and other viruses. This strategy has been successfully employed for several viruses. *Retroviridae* has an assembly mechanism similar to the model of Ty1 and is thus an appropriate choice for CTVI antiviral studies. Because of this specific principle, CTVI also has great potential for the development of preventive vaccines.

## 3. Application of CTVI for Different Viral Classes

### 3.1. Retroviruses

#### 3.1.1. Murine Leukaemia Virus

Murine leukaemia virus (MLV) is an RNA tumour virus that causes active leukaemia in mice. The polyprotein encoded by the virus is processed into several proteins, mainly the capsid protein (P27), matrix protein (P19), nucleocapsid protein (P12) [40], envelope protein (gp85), and transmembrane protein (gp37). MLV exists as an exogenous ecotropic virus or an endogenous retrovirus [41].

Natsoulis and Boeke (1991) first verified the feasibility of CTVI [3]. Subsequently, in 1995 Natsoulis [7] took advantage of the strategy using SN, which resulted in effectual inhibition of MLV multiplication, decreasing the production of progeny virus. In 1997, the *Escherichia coli* RNase HI enzyme was adopted by Schumann as a substitute for SN in CTVI [42]. Remarkably, RNase HI was found to be nontoxic to the host cell and had a highly efficient antiretroviral effect when fused to the C-terminus of the viral group-specific antigen (Gag) protein [43]. The fusion protein with enzymatic activity was stably expressed in virus-susceptible cells. In the same year, Vanbrocklin [8] for the first time utilized RNase HI for MLV, and the enzyme exhibited a strong antiviral effect by inhibiting viral replication. In 2000, Vanbrocklin [24] sought to thoroughly determine the limits of CTVI with regard to viral and cellular factors in cultured cells. Eventually, the author found that the antiviral effector could be suggested by the number of cells in a different generation that expressed the fusion proteins; for example, DF-1 cells showed a chronic therapeutic efficacy and higher expression of fusion proteins compared with chick embryo fibroblasts (CEFs) [44,45,46]. Additionally, a range of poultry cells lacked permanent expression for disrupting endogenous and exogenous viruses [18]. Thus, certain poultry cells appear to create difficulty in some experimental systems. In contrast, the adherent, nontransformed EV-O-derived cell line DF-1 [47] supported good growth and efficient replication of avian sarcoma leukosis virus (ALV) or MLV [48] as well as the expression of foreign genes. Thus, DF-1 cells were found to be suitable for long-term research. Moreover, with regard to the extent of the enzyme’s activity, RNase HI identifies an RNA–DNA mixture [49,50] and degrades only double-stranded RNA (dsRNA) [51]. However, RNA–DNA is an intermediate in retrovirus replication. SN, an enzyme secreted by *S. aureus* and a multipurpose nuclease, can destroy both DNA and RNA, regardless of its single-stranded or double-stranded nature. However, SN requires Ca^2+^ for activity and cleaves extracellular nucleic acids. More importantly, both RNase HI and SN lack detectable cell toxicity [42]. Thus, it appears that SN is a relevant restrictive element for CTVI. Researchers expect that this strategy can be applied to other retroviruses and that this approach may be broadly applicable to other virus families.

#### 3.1.2. Classical Swine Fever Virus

CSFV, together with bovine viral diarrhoea virus (BVDV) [52,53,54] and border disease virus (BDV) of sheep [55,56], are members of the genus *Pestivirus* within the family *Flaviviridae*. CSFV is an enveloped RNA virus that mainly causes highly contagious, lethal, classical swine fever in pigs, with enormous economic losses in animal husbandry [57,58,59]. At present, there are few attenuated live vaccines or highly effective drugs available to protect against or to efficiently treat CSFV infections. Existing vaccines have drawbacks such as interference with serodiagnosis, i.e., they mask virus positivity. Thus, a method for preventing infection of this virus is urgently needed.

In 2010, sufficient experimental evidence at the level of intracellular immunization was consecutively used to demonstrate the antiviral effects of capsid–SN, which were separately derived from the CSFV capsid protein and the nuclease of *S. aureus* by Wang [60] and Zhou [10]. The capsid–SN fusion protein was also found to be incorporated into virions during the viral replication cycle of integration and assembly. The capsid–SN fusion disrupted the genome of CSFV in the extracellular environment and virtually abolished the infectivity of progeny virions. Therefore, CTVI may be a novel antiviral approach for controlling CSFV infection.

#### 3.1.3. Human Immunodeficiency Virus-1

Acquired immunodeficiency syndrome (AIDS) is a severe, transmissible, fatal disease of humans caused by HIV-1. HIV-1, a retrovirus of the *Lentivirus* genus with an extremely long incubation period, attacks the human immune system, targeting highly important T4 leucomonocytes [61,62].

**Figure 2:**
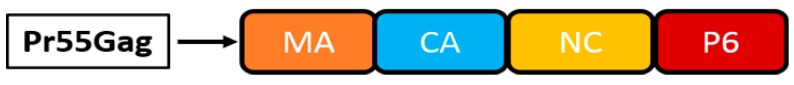
Gag protein components. The structural protein Gag is mainly processed into four proteins, p17 matrix (MA), p24 capsid (CA), p9 nucleocapsid (NC), and P6, by a virus-encoded protease.

Together with HIV-2, HIV-1 originated from the midwestern region of Africa and was transmitted from primates to humans. The viral genome consists of double positive-sense strand RNA, which harbours long terminal repeats (LTRs) at both ends. At least nine proteins, categorized into structural proteins (Gag, Pol, envelope (Env)), regulatory proteins (trans-activator of transcription (Tat), regulator of expression of virion proteins (Rev)), and accessory proteins (virus protein U (Vpu), viral protein R (Vpr), viral infectivity factor (Vif), negative regulatory factor (Nef)), are encoded by the sequence between the LTRs. The Gag gene is translated into a 55 kD protein (pr55), which is processed into p17 matrix (MA), p24 capsid (CA), p9 nucleocapsid (NC), and p6 by a virus-encoded protease (Figure 2). The fusion protein Gag–Pol is generated by ribosomal frameshifting caused by a *cis*-acting motif in the mRNA [63]. The interior of the capsid contains the genomic RNA, enzymes (reverse transcriptase, integrase [64,65], and protease), as well as host cell components.

To date, some vaccines against HIV-1 [66,67,68,69] have been applied, and some effects of early antiretroviral therapy have been achieved [70,71]. In 1995, Wu [22] examined HIV-1 Vpr (Vpr1) and HIV-2 (Vpx2) to determine whether it was possible to target to the HIV particle foreign proteins carrying an SN/SN* mutation. In 1998, Kobinger [21] developed Vpr-based fusion proteins that can specifically incorporate into the mature virion, impacting the structural and functional integrity of the normal virus. In addition, Okui [20] constructed a scAb and HIV-1 Vpr fusion that inhibited HIV-1 integrase (IN) [72], protease(PR), and reverse transcriptase(RT). The scAb portion binds specifically to residues 228 to 235 of HIV-IN, inhibiting IN in vitro and blocking viral replication in vivo when expressed intracellularly before infection [39,73]. Targeting of IN, a major enzyme that plays a critical role in infection in the early stage of the retroviral life cycle [74] and is a C-terminal section of Gag–Pol polyprotein [75], is interesting because it can influence reverse transcription [76,77,78,79]. Vpr, which plays a distinct and necessary role in viral replication and pathogenesis, is a virion-associated auxiliary protein of approximately 100 amino acids [80]. The fusion protein scAbE–Vpr was found to be incorporated into virions during viral assembly, forming virus-like particles with an element deleterious to the viral component, and chiefly targeted HIV-1 IN by interfering with its folding. Hence, scAbs may be a preferred candidate for CTVI strategies. As evaluated in this assay, an oligopeptide was found to be the most suitable fusion partner because of the nearly complete removal of virus infectivity—a reduction of over 10^3^-fold [81].

### 3.2. Flavivirus

#### 3.2.1. Dengue 2 Virus

DENV, which can be transmitted by most mosquitos, is a member of *Flavivirus* and a serious public health issue, mainly in tropical and subtropical areas. DENV, an enveloped single-stranded positive-sense RNA virus, primarily includes four serotypes (DENV1–DENV4) [82,83]. The genome of DENV is approximately 10.5 kb and consists of a single continuous open reading frame (ORF). The ORF encodes a polyprotein precursor that is processed into three structural proteins (capsid, membrane, and envelope) and seven nonstructural proteins (NS1, NS2A, NS2B, NS3, NS4A, NS4B, NS5) by viral and host proteases to produce the mature viral proteins [84,85]. In the mature viral particle, the multicopy capsid protein is mainly responsible for enveloping the single-copy nucleic acids of the virus, forming a protein complex termed the nucleocapsid. Based on previous research in 2004, Qin [16,86] first suggested that CTVI may be associated with resistance to DENV, and an experiment was designed to verify his hypothesis. To establish the prophylactic model, the Ca^2+^-dependent nuclease SN was fused to the C-terminus of the capsid protein of DENV. The fusion gene capsid–SN was then ligated to a eucaryotic plasmid, pcDNA6/V5–His, to produce the recombinant plasmid pcDNA6/V5–His–capsid–SN. This plasmid was transfected into BHK-21 cells, and blasticidin was used to select cell lines stably expressing the fusion protein. After infection of the cell lines with DENV, the viral titres in some proportion of the experimental group were markedly lower than those of the control group. This exploratory experiment confirmed that the fusion protein stably expressed by the cell line possessed nuclease activity but was not cytotoxic. The result suggested an effective experimental system for CTVI as well as a preventive antiviral construct. Some researchers have thoroughly investigated the therapeutic effects of CTVI to DENV in vitro [17], and others have reported the assembly of nucleocapsid-like particles (NLPs). These studies on DENV may provide an excellent reference for a candidate vaccine [87].

#### 3.2.2. Japanese Encephalitis Virus

JEV, also belonging to the genus *Flavivirus*, exhibits a structure analogous to that of DENV [88,89]. JEV is a mosquito-transmitted virus that is distributed internationally, mainly in India, Southeast Asia, and China, and causes serious nervous diseases and critical morbidity and mortality [90] in both humans and animals.

DNA vaccines involving mutation of assumed *N*-linked glycosylation sites of JEV, membrane (prM) and envelope (E) proteins [23,91], RNA interference [92], and an attenuated live vaccine (SA14-14-2 strain) [93] with unexceptionable humoral and cytotoxic cellular immune responses have been extensively applied in humans and animals, albeit not without adverse effects. In the absence of effective therapeutic drugs, the virus still has a substantial influence on human and veterinary health. Based on the prior effectiveness of CTVI, Pang [12] made the first attempt using this strategy for JEV, ultimately confirming that CTVI is an efficient antiviral method. Currently, investigators are establishing an animal model to evaluate the effect of antiviral therapy in vivo, which may further the application of CTVI.

### 3.3. Hepadnaviruses

HBV, a major aetiologic agent of liver cirrhosis and hepatocellular carcinoma, is a major public health problem globally, in both humans and animals, particularly in Asian and African countries [94,95]. The illness has a substantial economic burden and is associated with severe illness and death. Thus, efficient treatment measures are urgently needed, and many different treatment methods are being investigated to combat the disease. Thus far, interferon-α (IFN-α) [96,97] is the first choice for treating HBV infection, but it lacks the desired curative effect. Other anti-HBV therapies, such as immunomodulator and emulative reverse transcriptase inhibitors, mainly suppress replication of the genome [98]; some vaccines based on recombinant surface antigens of HBV with higher immunogenic power have also attracted much attention. Despite the current success, investigators have not stopped to explore new antiviral approaches, such as scAbs, against HBV core proteins.

The HBV virion consists of an outer lipid envelope and icosahedral nucleocapsid core. The relaxed circular DNA (rcDNA) genome facilitates the reverse transcription reaction of HBV pregenomic RNA (pgRNA) [98], which is encompassed by viral core proteins and DNA polymerase, forming the nucleocapsid core (NC). The reverse transcription and positive-strand DNA synthesis reactions proceed in the NC, completing replication of the viral genome. Thus, although HBV is a DNA virus, it has reverse transcriptase activity analogous to that of retroviruses. Beterams [14] and Liu [13] took advantage of the viral core protein, which specifically identifies and packages HBV pgRNA, as a targeting molecule and ribonuclease, which combines and cleaves pgRNA, as an effector molecule. According to the results, the nuclease possesses the capacity to significantly inhibit replication in cultured cells, which may illustrate the applicability of CTVI.

## 4. Discussion

Although CTVI has been extensively applied, there are several issues that remain to be resolved. First, few of the nucleases that are appropriate for CTVI can be tolerated by the host cell without conspicuous toxicity. Therefore, it is necessary to explore other types of fusion partners that are not only nontoxic to the host cell but that can also efficiently inactivate the virion from within, such as SAMHD1 [99]. Strikingly, a more efficient antiviral effect may be achieved for some viruses which have a protein that can interact with scAbs, if fusion to both an enzyme and scAb is performed, combining both mechanisms to interfere not only with the viral genome but also with viral proteins to block the production of infective virus. Although a promising hypothesis, this has not been reported to date.

The CTVI strategy is currently at the in vitro cellular level of development. It is obvious that additional animal experiments are potentially important for elucidating the mechanisms or effects in vivo. Moreover, both in vivo and in vitro, it is difficult to guarantee that the recombinant plasmids transfected into cells or injected into the body will not be intercepted and removed by the immune system. Additionally, use of the CTVI approach will require that the plasmids enter cells efficiently and are stably expressed. Thus, it is necessary to screen cells in vitro using appropriate antibiotics such as G418 to ensure that the exogenous DNA is integrated into the chromosome of the host cells for efficient replication and stable expression of the fusion proteins. Intracellular expression of the foreign molecule allows access to the viral nucleic acid or protein. Alternatively, the effects may depend on the efficiency of gene transfer, which may solve the interference between the recombinant plasmid and the immune system and host immune responses to the effector molecules, as well as, the copy number of the molecule within cells.

## 5. Conclusions

Altogether, convincing evidence has been provided through the successful application of CTVI to the viruses mentioned above. CTVI, a new antiviral approach that virtually eliminates progeny virus, has both prophylactic and therapeutic antiviral effects and has potential applications against viruses with relatively flexible capsid structures, such as *Retrovirus*, *Paramyxovirus* and *Hepadnavirus*. Presently, CTVI is also used in *Reoviridae* for transgenic construction [100]. Accordingly, a thorough understanding of CTVI and its possibilities may provide additional insight into the progress of antiviral studies and reveal possible candidates for preventive and therapeutic vaccines. Furthermore, the CTVI experimental system may be of use in the development of antiviral medicines and perhaps other fields.

## Figures and Tables

**Table 1:** Application of CTVI for different types of viruses.

Viral Type	Virus	Genome	Viral Protein	Foreign Molecule	Fusion Protein	Location of Enzyme	Plasmid Vector	Cell	Antibiotic	References
Retroviruses	Mo-MLV	RNA	Gag/Gag-Pol	SN/RNase HI	Gag-SN/Gag-RNase HI	C-terminus	pGN1600	RCASBP		[18]
	CSFV	RNA	Capsid	SN	Capsid-SN	C-terminus	pcDNA	PK-15	G418	[19]
	HIV-1	RNA	Vpr	scAb	Vpr-scAb	C-terminus	pCXN2	293T	G418	[20,21]
			Vpr	SN	Vpr-SN	C-terminus	pLR2P	HeLa		[22]
Flavivirus	JEV	RNA	Capsid	SN	Capsid-SN	C-terminus	pcDNA3.1	BHK-21		[12,23]
	DENV2	RNA	Capsid	SN	Capsid-SN	C-terminus	pcDNA6/V5-His	BHK-21	blasticidin	[16,17,24]
Circovirus	PCV2	DNA	Capsid	SN	Capsid-SN	C/N-terminus	pIRESneo	PK15	G418	
Hepadnaviruses	HBV	DNA	Capsid	Ribonuclease	p/TN	C-terminus	pcDNA3.1 (−)	HepG2.2.15		[13]
			Capsid	SN	Capsid-SN	C-terminus	pcDNA6/Myc-His	Huh7		[14,25]

SN: staphylococcal nuclease; Gag: group-specific antigen; Pol: polymerase; Vpr: viral protein R; scAb: single-chain antibody; Moloney murine leukaemia virus (Mo-MLV); classical swine fever virus (CSFV), human immunodeficiency virus-1 (HIV-1); Japanese encephalitis virus (JEV); hepatitis B virus (HBV); dengue 2 virus (DENV2).
